# Functional Gene Polymorphism to Reveal Species History: The Case of the *CRTISO* Gene in Cultivated Carrots

**DOI:** 10.1371/journal.pone.0070801

**Published:** 2013-08-05

**Authors:** Vanessa Soufflet-Freslon, Matthieu Jourdan, Jérémy Clotault, Sébastien Huet, Mathilde Briard, Didier Peltier, Emmanuel Geoffriau

**Affiliations:** 1 Agrocampus Ouest, UMR1345 Institut de Recherche en Horticulture et Semences, SFR4207 QUASAV, Angers, France; 2 Université d’Angers, UMR1345 Institut de Recherche en Horticulture et Semences, SFR4207 QUASAV, Angers, France; 3 INRA, UMR1345 Institut de Recherche en Horticulture et Semences, SFR4207 QUASAV, Beaucouzé, France; University of New England, Australia

## Abstract

**Background:**

Carrot is a vegetable cultivated worldwide for the consumption of its root. Historical data indicate that root colour has been differentially selected over time and according to geographical areas. Root pigmentation depends on the relative proportion of different carotenoids for the white, yellow, orange and red types but only internally for the purple one. The genetic control for root carotenoid content might be partially associated with carotenoid biosynthetic genes. Carotenoid isomerase (*CRTISO*) has emerged as a regulatory step in the carotenoid biosynthesis pathway and could be a good candidate to show how a metabolic pathway gene reflects a species genetic history.

**Methodology/Principal Findings:**

In this study, the nucleotide polymorphism and the linkage disequilibrium among the complete *CRTISO* sequence, and the deviation from neutral expectation were analysed by considering population subdivision revealed with 17 microsatellite markers. A sample of 39 accessions, which represented different geographical origins and root colours, was used. Cultivated carrot was divided into two genetic groups: one from Middle East and Asia (Eastern group), and another one mainly from Europe (Western group). The Western and Eastern genetic groups were suggested to be differentially affected by selection: a signature of balancing selection was detected within the first group whereas the second one showed no selection. A focus on orange-rooted carrots revealed that cultivars cultivated in Asia were mainly assigned to the Western group but showed *CRTISO* haplotypes common to Eastern carrots.

**Conclusion:**

The carotenoid pathway *CRTISO* gene data proved to be complementary to neutral markers in order to bring critical insight in the cultivated carrot history. We confirmed the occurrence of two migration events since domestication. Our results showed a European background in material from Japan and Central Asia. While confirming the introduction of European carrots in Japanese resources, the history of Central Asia material remains unclear.

## Introduction

Selection events along the species or breeding history generally lead to signatures of selection at the molecular level, i.e. local variations of diversity or allele frequencies at genes underlying phenotypic variations or in their surrounding region. The detection of signatures of selection encounters various issues. Firstly, demographic events like population subdivision can modify genetic polymorphism and mimic selective events [1]. Secondly, selection pattern can vary along a gene [2], [3]. Signatures of selection are therefore detected or not, according to the targeted genomic region and the linkage disequilibrium (LD; i.e., the non-random association of alleles at different loci) throughout the gene.

The history of carrot (*Daucus carota* subsp. *sativus*), cultivated worldwide, might have lead to such selection signatures. The domestication of this species is thought to have occurred in the Afghanistan region before the 900 s [4]. The first cultivated carrots were purple or yellow rooted. Their cultivation spread along trade routes, reaching Middle East and North Africa, and then to Europe in the Middle Ages. They were gradually replaced by white- and orange-rooted forms, which appeared in the 1600 s [5]. Concomitantly, a red type appeared in Asia, and particularly in Japan in the 1700 s [6], [7]. Finally, since the nineteenth century, orange-rooted carrots have spread from Europe to other continents and have become predominant commercially. Diversity analyses were recently assessed in cultivated carrot and revealed a genetic subdivision between Western (European and American) and Eastern (Asian) accessions [8], [9].

Root pigmentation depends on the relative proportion of different carotenoids, in combination with anthocyanins for the purple type [10]. Orange- and red-rooted carrots accumulate large amounts of carotenoids, mainly β- and α-carotene for the orange type or lycopene and β-carotene for the red type. Yellow-rooted carrots present low amounts of carotenoids, especially lutein and β-carotene. White-rooted carrots contain negligible amounts of carotenoids. The genetic control for root carotenoid content can be associated with carotenoid biosynthetic genes in different species including carrot [11], [12], [13]. These data suggest that the carotenoid biosynthetic genes may have been targeted by human selection.

The carotenoid biosynthesis pathway was established in higher plants [14], [15]. This pathway involves a series of desaturations, cyclisations, hydroxylations and epoxidations. Most of the carotenoid genes were identified in various species [14], [15], [16], [17], [18] including carrot [19].

Among genes, the carotenoid isomerase (*CRTISO*) has emerged as a regulatory step in the carotenoid biosynthesis pathway. CRTISO enzyme catalyses the *cis*-to-*trans* isomerisation reactions leading to all *trans*-lycopene, the substrate for the subsequent lycopene cyclisation to form all *trans*-*α*/*β*-carotene [20]. The identification of *CRTISO* as an isomerase was also confirmed by its functional expression in *Escherichia coli* in which it was able to convert *cis*-carotenoids to all *trans*-carotenoids [17], [18]. *CRTISO* mutants, such as *ccr2* (*Arabidopsis*), *phs3* (rice) and *tangerine* (tomato), result in the accumulation of *cis*-carotenoids in the dark-grown plants [17], [21] and fruits [18].

During plant evolution, paralogous genes for several carotenoid biosynthesis enzymes have been conserved and subjected to subfunctionalization: the paralogs are differentially expressed in photosynthetic or non-photosynthetic tissues [22]. A mutation affecting the paralog expressed in non-photosynthetic tissues would therefore not affect the carotenogenesis needed for photosynthesis. In most species in which carotenoid isomerase was characterized (except maize, [23]), only one gene was found (tomato [17], carrot [19], rice [24]). One hypothesis to this tendency of *CRTISO* to be single-copy is that CRTISO activity could be partially redundant in the light because of photoisomerisation. Indeed, light could substitute for the lack of isomerase activity in *ccr2* mutants which then synthesize efficiently most carotenoids [18]. A similar light-induced isomerisation of prolycopene to all *trans*-lycopene was observed in the outer green tissues in immature fruit of *tangerine* mutants that were exposed to light but not in non-photosynthetic tissues of flowers (petals), ripe fruit, and the innermost parts of the green fruit [17]. Consequently, the function of *CRTISO* in plants is presumably to enable carotenoid biosynthesis to occur in the dark and in non-photosynthetic tissues such as the root, case of carrot. By comparison to other crucial steps in the carotenoid pathway, *CRTISO* may have evolved with fewer constraints due to photoisomerisation, and may have been more prone to be subjected to artificial selection in non-photosynthetic organs, like root. Clotault et al. [25] showed that *CRTISO* gene has undergone through selection events in cultivated carrot but the polymorphism pattern was observed among partial *CRTISO* sequence (only 700–1,000 pb). The particular status of this gene and preliminary results suggest that *CRTISO* gene could be a good candidate for selection signature research. The analysis of the nucleotide polymorphism and the LD among the complete *CRTISO* sequence will enable to clarify the selection pattern, depending on the gene structure and in relation with colour types. Moreover, selection effect can spread over genomic regions around the gene by a selective sweep. The study of the intrachromosomic LD will allow testing this hypothesis.

Our work aimed to know whether polymorphism within *CRTISO*, a key gene involved in the carotenoid biosynthesis pathway especially in root organs, should reflect the cultivated carrot history, including selective events during breeding. The present results showed a signature of selection pattern at the *CRTISO* gene and the critical contribution of *CRTISO* polymorphism for explaining the cultivated carrot history, especially regarding material from Asia and Central Asia.

## Materials and Methods

### Plant Material

Thirty nine cultivars of carrot (*Daucus carota* subsp. *sativus*) were sampled (Table 1); each one was represented by a single individual. These cultivars were chosen to maximize the diversity according to geographical origin, root colour and shape. Seeds were obtained from several seed banks and breeding companies. Genomic DNA was extracted from 50 mg of young freeze-dried leaves by using a modified CTAB protocol [26].

**Table 1:** List of carrot cultivar samples.

Code	Cultivar name	Root colour	Geographical origin	Source^a^	*CRTISO* haplotypes^b^
100	Kuttinger	White	Switzerland	ACO-IRHS	1/1
104	Blanche Collet Vert Hors Terre	White	France	ACO-IRHS	2/2
105	Blanche des Vosges	White	France	ACO-IRHS	2/2
108	Long White Green Top	White	Denmark	WGRU	2/2
109	White Belgian	White	United Kingdom	WGRU	3/3
112	SAR112	White	Middle East	ACO-IRHS	6/13
208	Gelbe Lobbereicher	Yellow	Germany	WGRU	2/2
210	Jaune du Doubs	Yellow	France	ACO-IRHS	2/2
222	Golden Promise	Yellow	Asia	Mikado-Kyowa Seeds	4/4
223	Lobberich	Yellow	Italy	ACO-IRHS	2/2
224	Ch-Wy	Yellow	Asia	Mikado-Kyowa Seeds	6/14
226	YC226	Yellow	Asia	ACO-IRHS	4/4
230	Taborska	Yellow	Czech Republic	Nohel Garden	2/2
231	Shima Ninjin	Yellow	Japan	Mikado-Kyowa Seeds	6/6
307	Kuroda	Orange	Japan	ACO-IRHS	9/10
313	Amsterdam 2 Sweetheart	Orange	Netherlands	WGRU	2/2
337	De Colmar à Cœur Rouge 2	Orange	France	ACO-IRHS	2/2
360	Flakkee	Orange	Netherlands	ACO-IRHS	1/1
365	Mestnaya LR	Orange	Kyrgyzstan	VIR	15/15
366	Mestnaya Zheltaya	Orange	Uzbekistan	VIR	11/12
369	Mestnaya LR	Orange	Kazakhstan	VIR	11/11
370	Sapporo futo	Orange	Japan	Mikado-Kyowa Seeds	11/11
372	Koizumi Riso	Orange	Japan	Mikado-Kyowa Seeds	10/11
373	Kokubu Senso Oonaga	Orange	Japan	Mikado-Kyowa Seeds	11/11
374	Heian -3 Sun	Orange	Japan	Mikado-Kyowa Seeds	2/2
3015	Nantaise améliorée	Orange	France	ACO-IRHS	2/2
403	Annual Red Rawalpindi	Red	Pakistan	WGRU	5/5
407	Pink Selection	Red	China	WGRU	5/5
411	Red Queen	Red	India	Sungro	5/5
420	Pusa Kesar	Red	India	WGRU	6/6
421	JF421	Red	Asia	ACO-IRHS	6/6
426	Honbeni Kintoki	Red	Japan	Takii	2/2
500	Anthocyanee G	Purple	Middle East	ACO-IRHS	2/2
514	Afghan Purple	Purple	Afghanistan	WGRU	6/6
515	PT2 515	Purple	Middle East	ACO-IRHS	8/8
516	PD516	Purple	India	ACO-IRHS	6/6
520	PM520	Purple	Middle East	ACO-IRHS	6/6
521	PD521	Purple	Middle East	ACO-IRHS	5/5
522	PA522	Purple	Middle East	ACO-IRHS	7/8

a ACO-IRHS: Agrocampus Ouest - Institut de Recherche en Horticulture et Semences (France); WGRU: Warwick Genetic Resources Unit (United Kingdom); VIR: Vavilov Research Institute (Russia).

b *CRTISO* haplotypes were inferred by DnaSP 5.10 software for each diploid carrot cultivar.

### Microsatellite Genotyping

In order to study genetic structure of the sample, 17 microsatellite primer pairs (Table S1) were chosen based upon their reproducibility and coverage of the carrot genome [27]. Five microsatellite markers were located around *CRTISO* gene to investigate the linkage disequilibrium on the linkage group 4.

PCR reactions were performed in 20 µL volume with 10–50 ng genomic DNA, 1X PCR buffer, 0.2 mM dNTPs, 2 mM MgCl_2_, 0.25 µM of fluorescent dye-labelled forward primer, 0.25 µM of reverse primer, and 1U Taq DNA polymerase (Interchim, Montluçon, France). Amplifications were carried out by using a MyCycler thermalcycler (Biorad, Hercules, CA). The thermalcycler was programmed as follows: initial denaturation at 94°C for 2 min, 35 cycles at 94°C for 30 s, annealing temperature for 30 s, 72°C for 30 s, and a final extension at 72°C for 10 min.

The amplified fragments were analyzed by capillary electrophoresis (ABI 3730 DNA Analyzer, Applied Biosystems, Foster City, CA), and labelled PCR products were automatically sized with Genemapper 3.7 software (Applied Biosystems, Foster City, CA).

### Genetic Structure

Microsatellite data were used to investigate the genetic structure of the sample with STRUCTURE 2.1 software [28]. The genetic model was used without information on the origin of each individual and allowing for admixture and allele correlated frequencies. Ten independent simulations, with a burn-in period length of 10^5^ and a run length of 10^6^, were used for each number of clusters (K) from one to ten. The most likely number of genetic clusters was selected using Evanno’s [29] and the highest *ln*P(P) methods. For each individual, the proportion *q* of its genome assigned to each cluster and its 95% confidence interval were calculated. An individual was assigned to a cluster if *q*>0.5. A CA (Correspondence Analysis) was also performed by using Genetix 4.05 software [30].

Fixation indexes were estimated among the colour groups (*F*_CT_), by using FSTAT 2.9 software [31] according to Weir and Cockerham (1984) formula [32].

### Allele Sequencing

**Figure 1:**
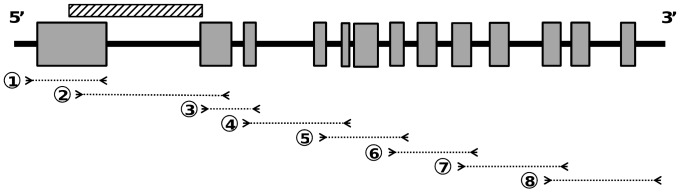
Position of primers used for *CRTISO* amplification. Boxes and lines represent exons and introns, respectively, of the *CRTISO* sequence. Arrows indicate partial sequences amplified with primers. The hatched box above the *CRTISO* sequence corresponds to the partial sequence analysed by [25].

Gene-specific primers were designed and synthesized (Figure 1), based on the mRNA sequence of *Daucus carota* subsp. *sativus* (GenBank accession number DQ192188) in order to obtain the complete *CRTISO* sequence (exons and introns).

PCR reactions were performed in 25 µL volume with 10–50 ng genomic DNA, 1X PCR buffer, 2 mM MgCl_2_, 0.2 mM dNTPs, 0.2 µM of both forward and reverse primers, and 1U Taq DNA polymerase (Interchim, Montluçon, France). Amplifications were carried out by using a MyCycler thermalcycler (Biorad, Hercules, CA). The cycling conditions were: initial denaturation at 94°C for 3 min, 35 cycles at 94°C for 30 s, annealing at 55°C for 45 s, 72°C for 2 min, and a final extension at 72°C for 5 min.

PCR products were purified with ExoSAP-IT (GE Healthcare, France) and sequenced with BigDye® Terminator 3.1 chemistry (Applied Biosystems, Weiterstadt, Germany) in an ABI Prism 3730 sequencer (Applied Biosystems, Weiterstadt, Germany) according to the supplier’s instructions. The primers used to amplify PCR fragments were also used for the sequencing. Eight partial genomic regions were sequenced with forward and reverse primers to discard PCR errors (Table 2). Nucleotide sequences were read with Sequence Scanner 1.0 software (Applied Biosystems, Weiterstadt, Germany) and the credibility between the two reads was checked with Geneious 5.0.2 software [33]. For each individual, the eight partially overlapping sequences were assembled in a contiguous sequence with a program compiled in C++ [34].

**Table 2:** Sequences of primers used for *CRTISO* amplification.

Amplified fragment		Primer sequence (5'–3')		Length (bp)
<$>\raster(65%)="rg1"<$>	5'UTR - Exon1	F	aatcaccttcctccccaaag	20
		R	tcactgaagccaaacatcaca	21
<$>\raster(65%)="rg2"<$>	Exon1 - Exon2	F	tggtgggagctctggatatt	20
		R	aaatggacagtactggggtca	21
<$>\raster(65%)="rg3"<$>	Exon2 - Exon3	F	cttaacttgataactcaagcattgg	25
		R	cagtgttaggcattccataggc	22
<$>\raster(65%)="rg4"<$>	Exon3 - Exon5	F	tgccttgaactcattggaac	20
		R	tgcgttgatcattggtgtct	20
<$>\raster(65%)="rg5"<$>	Exon4 - Exon7	F	ctcaaaatgctggagacatagc	22
		R	tcccatctggtagcatttga	20
<$>\raster(65%)="rg6"<$>	Exon7 - Exon9	F	ctggcgaatggaaatgagat	20
		R	tcccttctggagctaatgatg	21
<$>\raster(65%)="rg7"<$>	Exon9 - Exon11	F	ttggtcaaatttagaggttcca	22
		R	ggcattcctagtaaaccctttg	22
<$>\raster(65%)="rg8"<$>	Exon11 - 3'UTR	F	agacacacaggcgctacctt	20
		R	caaccttctgcccttcatgt	20

F, forward primer; R, reverse primer.

The contiguous sequences of the 39 individuals were aligned with ClustalW software [35]. For heterozygous individuals, allelic phases were reconstructed by using the algorithm provided by PHASE implemented in DnaSP 5.10 software [36].

### Sequence Polymorphism

The population genetic parameters were defined by using DnaSP 5.10 software [36]. Sites with alignment gaps were excluded from analysis.

The population-level genetic variation was estimated as nucleotide polymorphism (*θ_w_*; [37]) and nucleotide diversity (*π*; [38]). *θ_w_* is based on the number of segregating sites while *π* is based on the pairwise differences between sequences in the population. The analyses of nucleotide polymorphism (*θ_w_*) and nucleotide diversity (*π_T_)* were based both upon the overall *CRTISO* sequence and upon a 100 bp window with a step size of 25 bp.

Haplotypes were inferred with DnaSP 5.10 software by including infinite-site violations. The number of haplotypes *h* and haplotype diversity *Hd* were then calculated [38].

A median-joining network establishing relationships among haplotypes based on the number of polymorphisms (each insertion/deletion -indel- was considered as a single nucleotide polymorphism -SNP-) was performed with Network 4.61 software [39].

### Linkage Disequilibrium

Linkage disequilibrium (LD) is the non-random association of alleles at different loci. The level of LD is influenced by many factors such as population subdivision, selection, genetic linkage, recombination rate [40]. We chose to measure LD by using *r^2^* because it summarizes both recombination and mutation histories [41].

LD was investigated by using TASSEL 3.0 software [42] not only along the complete *CRTISO* sequence but also along the linkage group 4 where *CRTISO* gene and five microsatellite markers were mapped [27]. Since rare alleles can generate a large variance in LD estimates, only biallelic loci with at least 5% frequency were used to calculate *r^2^*, which was plotted for all pairwise comparisons among SNPs. Given the influence of population subdivision, LD was calculated in each genetic group revealed by population structure analysis.

### Neutrality Tests

Tajima’s *D* test [43], implemented in DnaSP 5.10 software [36], was used to estimate the allele frequency departure from neutral expectation, by considering the dataset as a whole, but the genetic and colour groups separately. Positive and negative *D* values reveal an excess of intermediate and low frequency variants, respectively. Tajima’s *D* test was calculated for the overall *CRTISO* sequence and upon a 100 bp window with a step size of 25 bp. The statistical significance for the above tests was inferred provided that the observed value was included within the 95% confidence interval of neutral distribution, which was calculated by using 10,000 coalescent simulations in DnaSP by assuming no recombination.

The significance of observed Tajima’s *D* values was also compared to coalescent simulations according to a demographic model. This demographic model aimed at testing the role of genetic bottleneck during domestication and subsequent population subdivision during carrot cultivation. This model included two populations, corresponding to the Eastern and Western genetic groups, assuming constant effective population sizes, *N_E_* and *N_W_* respectively. At *T_d_* generations in the past, these two populations diverged. This divergence was confounded with the end of a genetic bottleneck, started at *T_b_*, and characterized by an effective population size *N_b_*. Before the bottleneck, the ancestral population size was *N_A_*. In domestication modeling studies, the bottleneck intensity *d* = *T_b_*–*T_d_* and *N_b_* are positively correlated and define the bottleneck severity *k* = *N_b_*/*d* [44]. We chose arbitrarily a fixed *d* value of 100 generations and varied *N_b_* in order to test ten *k* values: 0.2, 0.5, 1, 2, 5, 7, 10, 15, 20 and 50. Other model parameters were sampled from posterior parameter distribution obtained in [25], by using the algorithm described in [45]. For each *k* value, 5,000 coalescent simulations were made by using ms software [46]. For each simulation, 40 sequences from East and 38 sequences from West were obtained in order to mimic the most closely the biological dataset. Tajima’s *D* was calculated for each simulation by using Egglib [47]. The calculation for colour groups was made by sampling as many sequences from the Eastern and the Western groups as observed in each colour group. The statistical significance for Tajima’s *D* was inferred provided that the observed value was included within the 95% confidence interval of simulated distribution.

## Results

### Population Structure

**Figure 2:**
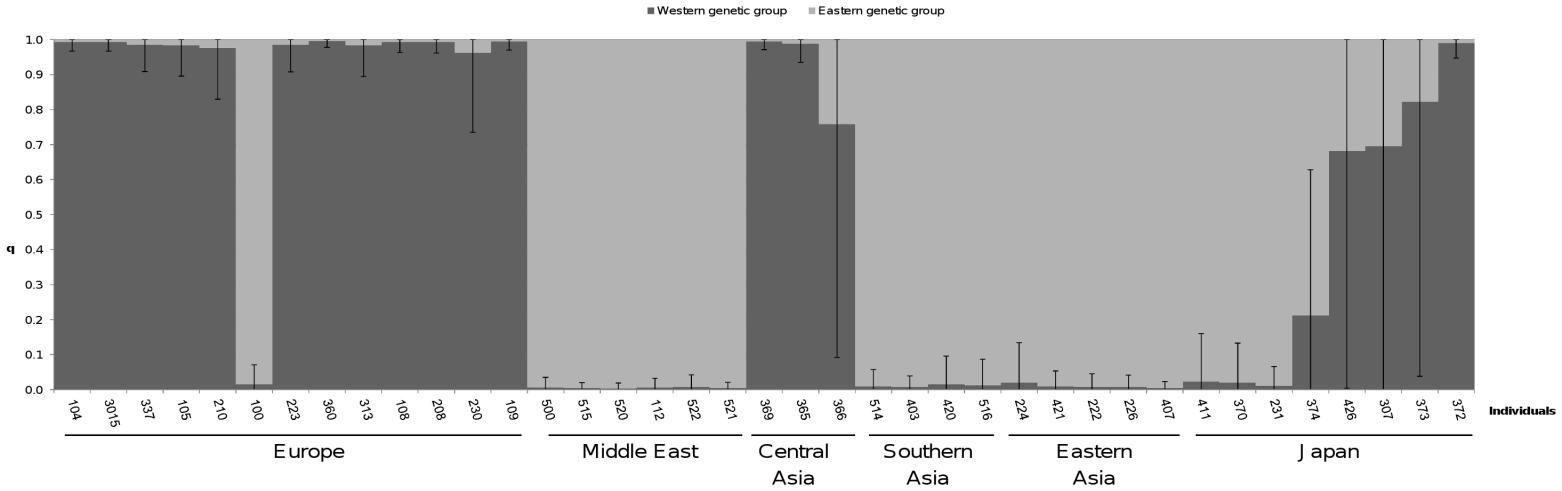
Genetic structure of 39 carrot cultivars based on a Bayesian approach on microsatellite data and assuming two clusters. Each individual is represented by a single vertical box broken in two coloured segments, with length proportional to each cluster assignment probability (*y*-axis). Bars represent the 95% confidence interval. The individuals are placed on the *x*-axis according to the approximate longitude of their country of origin, from France (left) to Japan (right).

All 17 microsatellite loci were polymorphic. A total of 174 alleles were identified with a mean of 10.2 alleles per locus, and a range from two to 19 alleles per locus. Delta K method (Figure S1) suggested two genetically distinct clusters as optimal, corroborated by CA analysis (Figure S2). But the log likelihood STRUCTURE analysis supported the presence of five clusters (Figure S1). Nevertheless, the subdivision of cultivated carrot into two genetic clusters is biologically meaningful and confirmed by literature [8], [9]. Among the studied 39 individuals, 34 were assigned to a cluster with a probability higher than 0.90 and five with a lower probability from 0.68 to 0.82 (Figure 2). The first cluster contained 19 individuals, including 12 from Europe, three from Central Asia and four from Japan. These individuals showed mainly orange, white or yellow roots. In accordance with the distinction from [8] and [9], this cluster was considered as the Western genetic group. However, the assignment probability in this cluster was the lowest for three orange rooted cultivars from Japan, along with high confidence interval, which showed an intermediate status of these cultivars. In contrast, two out of three Central Asia orange rooted cultivars were significantly assigned to this first cluster. The second cluster contained 20 individuals, only sampled in Middle East or Asia, except the Swiss cultivar ‘Kuttinger’ (code 100). Most of these individuals showed red, purple or yellow roots. This second cluster was considered as the Eastern genetic group.

Fixation indexes among colour groups (*F*_CT_) ranged from 0.003 to 0.142 (Table 3), showing the highest differentiation between orange and purple groups.

**Table 3:** Fixation indexes (*F*_CT_) among colour groups based on microsatellite data.

	Yellow	Orange	Red	Purple
**White**	0.019(NS)	0.049*	0.094*	0.114*
**Yellow**		0.003(NS)	0.015(NS)	0.057*
**Orange**			0.109*	0.142*
**Red**				0.053*

NS, non significant; *, *P*<0.05.

### Genomic Structure of the Carotenoid Isomerase in Carrot

The sequencing of *CRTISO* gene resulted in an alignment of 4,234 bp for 39 carrot diploid individuals.

The *CRTISO* gene in carrot exhibits the same genomic structure as other species (*Arabidopsis*, tomato, rice): these genes are split into 13 exons and 12 introns [17], [18], [24] (Figure 1). The coding and non-coding regions contributed respectively to 45 and 55% of the analysed sequences (excluding indels).

### Linkage Disequilibrium

**Figure 3:**
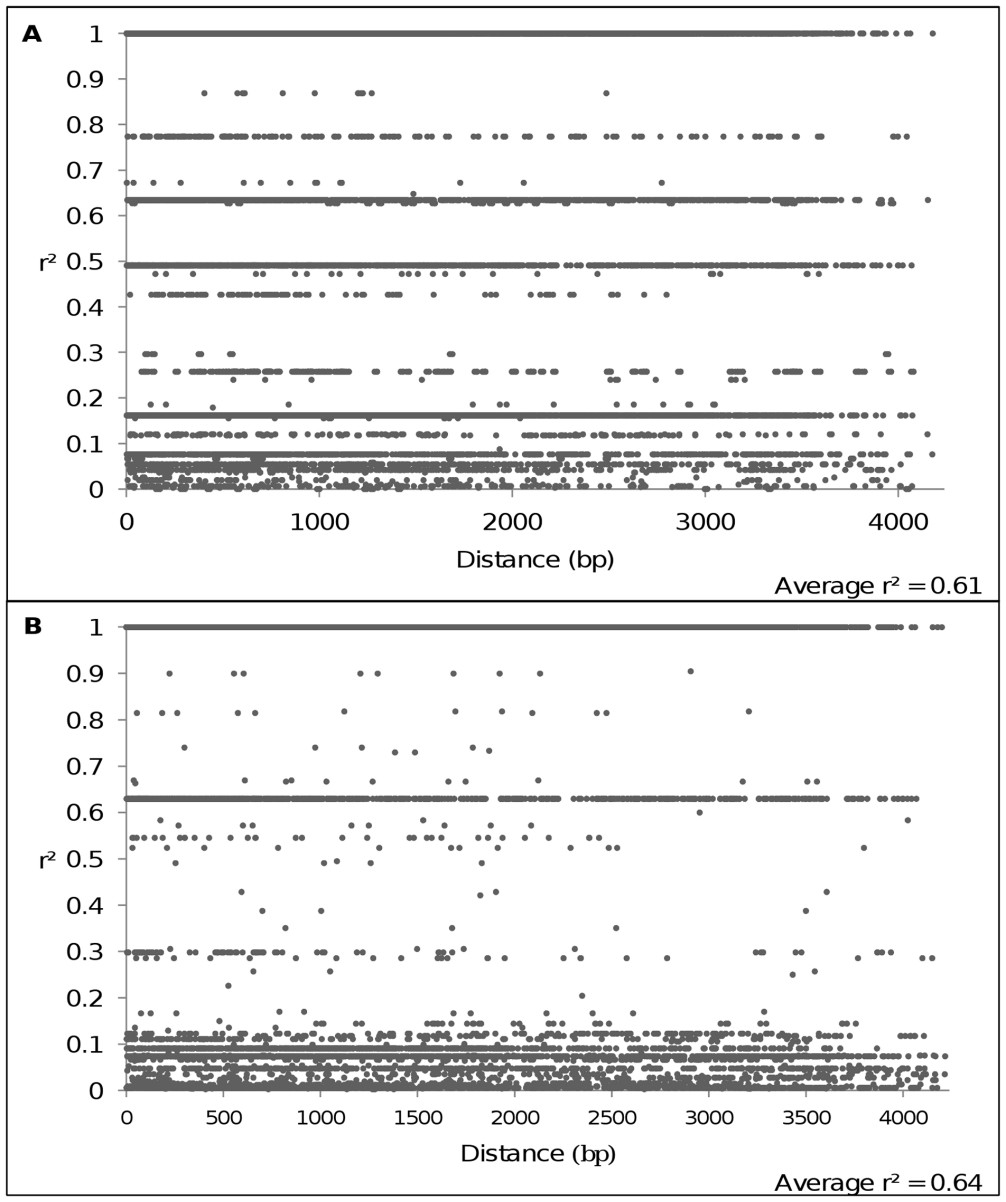
Linkage disequilibrium along *CRTISO* gene for the (A) Western and (B) Eastern genetic groups. LD level was measured by squared correlations of allele frequencies (*r^2^*) against physical distance between pairs of SNPs.

The complete *CRTISO* sequence exhibited a high LD level with an average *r^2^* of 0.61 for the Western genetic group and 0.64 for the Eastern genetic group. LD showed no decay within 4,234 bp (Figure 3), when tested with the method described in [48].

**Figure 4:**
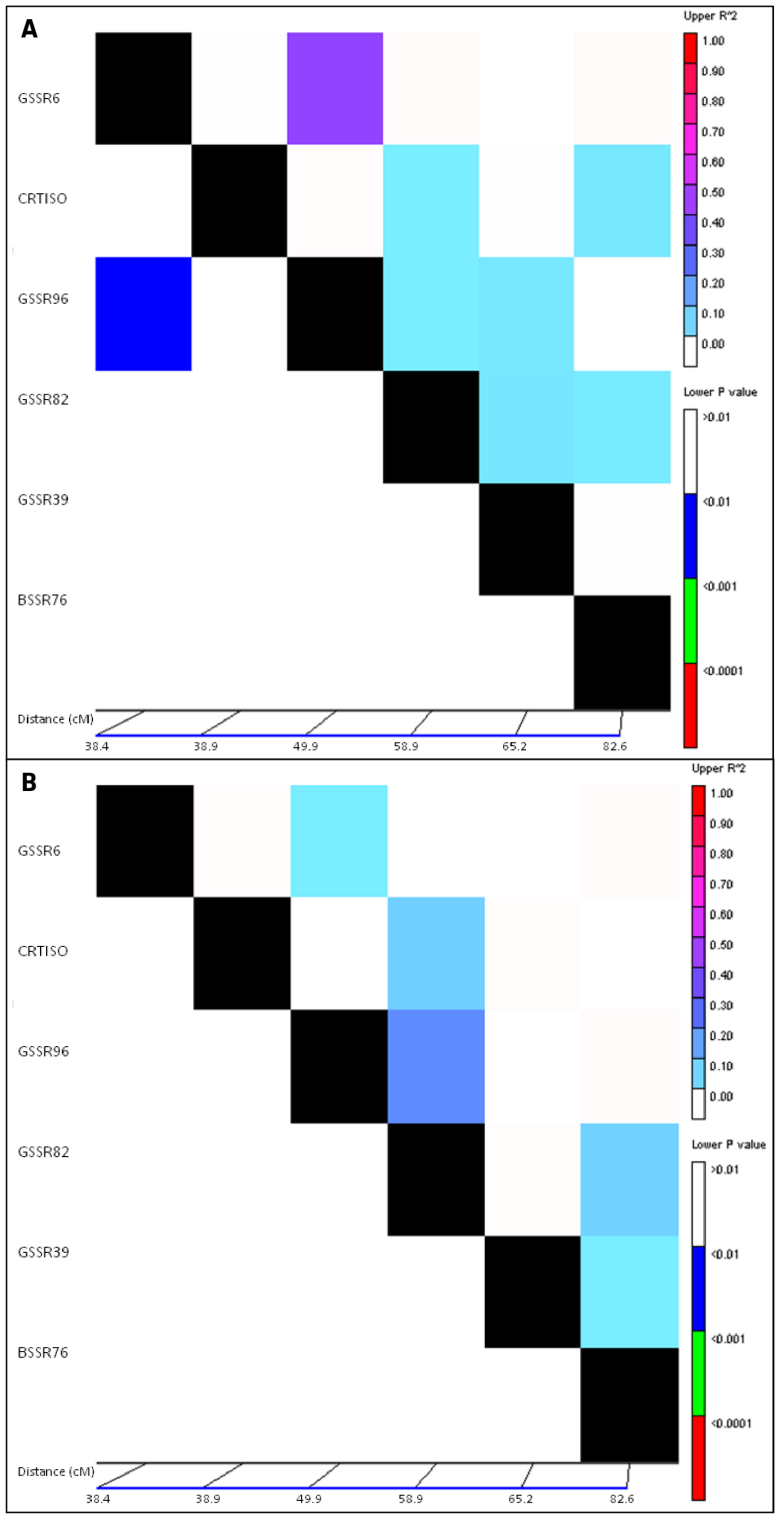
Linkage disequilibrium along linkage group 4 for the (A) Western and (B) Eastern genetic groups. LD level and its significance are represented at the top and at the bottom of the matrix, respectively. LD level was measured by squared correlations of allele frequencies (*r^2^*) against genetic distance. Significances of LD for microsatellite pairs are given as *P*-values determined by permutations. Each matrix compares the LD between pairs of markers displayed at the left and the bottom.

LD was also evaluated along LG4 [12] by using five microsatellite loci flanking *CRTISO* gene. Low LD level was observed between markers and *CRTISO* gene (Figure 4). Indeed, *r^2^* ranged from 0 to 0.24 for both Western and Eastern genetic groups. Only GSSR6 and GSSR96, separated by 11.5 cM, were in linkage disequilibrium (*r^2^ = *0.47) in the Western genetic group.

### Nucleotide Diversity and Neutrality Test

The observed nucleotide variation of the *CRTISO* genomic sequence, among the whole dataset, the Western versus Eastern genetic group, and the colour groups, is summarized in table 4.

**Table 4:** Nucleotide polymorphism of *CRTISO* in a sample of 39 carrot cultivars.

	No. of sequences	*θ_w_*	*π_T_*	*π_coding_*	*π_non-coding_*	*π_sil_*	*π_syn_*	*π_nonsyn_*	*π_nonsyn/_π_syn_*	*h*	*Hd*
**Total**	78	0.00973	0.01753	0.00928	0.02433	0.02496	0.02788	0.00354	0.12697	15	0.837
**West**	38	0.01018	0.01808	0.00912	0.02547	0.02591	0.02845	0.00335	0.11775	8	0.644
**East**	40	0.00971	0.00880	0.00479	0.01211	0.01242	0.01308	0.00201	0.15367	10	0.826
**White**	12	0.01462	0.02071	0.01099	0.02869	0.02952	0.03411	0.00409	0.11991	5	0.742
**Yellow**	16	0.01119	0.01915	0.00984	0.02681	0.02735	0.02947	0.0037	0.12555	4	0.692
**Orange**	24	0.01139	0.0176	0.00931	0.02444	0.02503	0.0283	0.00364	0.12862	7	0.786
**Red**	12	0.01222	0.0122	0.00603	0.01728	0.01757	0.01797	0.00209	0.11630	3	0.667
**Purple**	14	0.01191	0.01105	0.00541	0.01569	0.01598	0.01698	0.00177	0.10424	5	0.78

(Sequences without indels; total length 4,234 bp; coding region length 1,845 bp).

#### Within the whole dataset

Among the studied 78 allelic sequences, 15 haplotypes were identified, among which five were found only once (singleton haplotypes). Haplotypes differed by 212 polymorphisms, consisting of 196 nucleotide substitutions and 16 indels. Among the 55 substitutions found in coding regions, 18 were non-synonymous.

**Figure 5:**
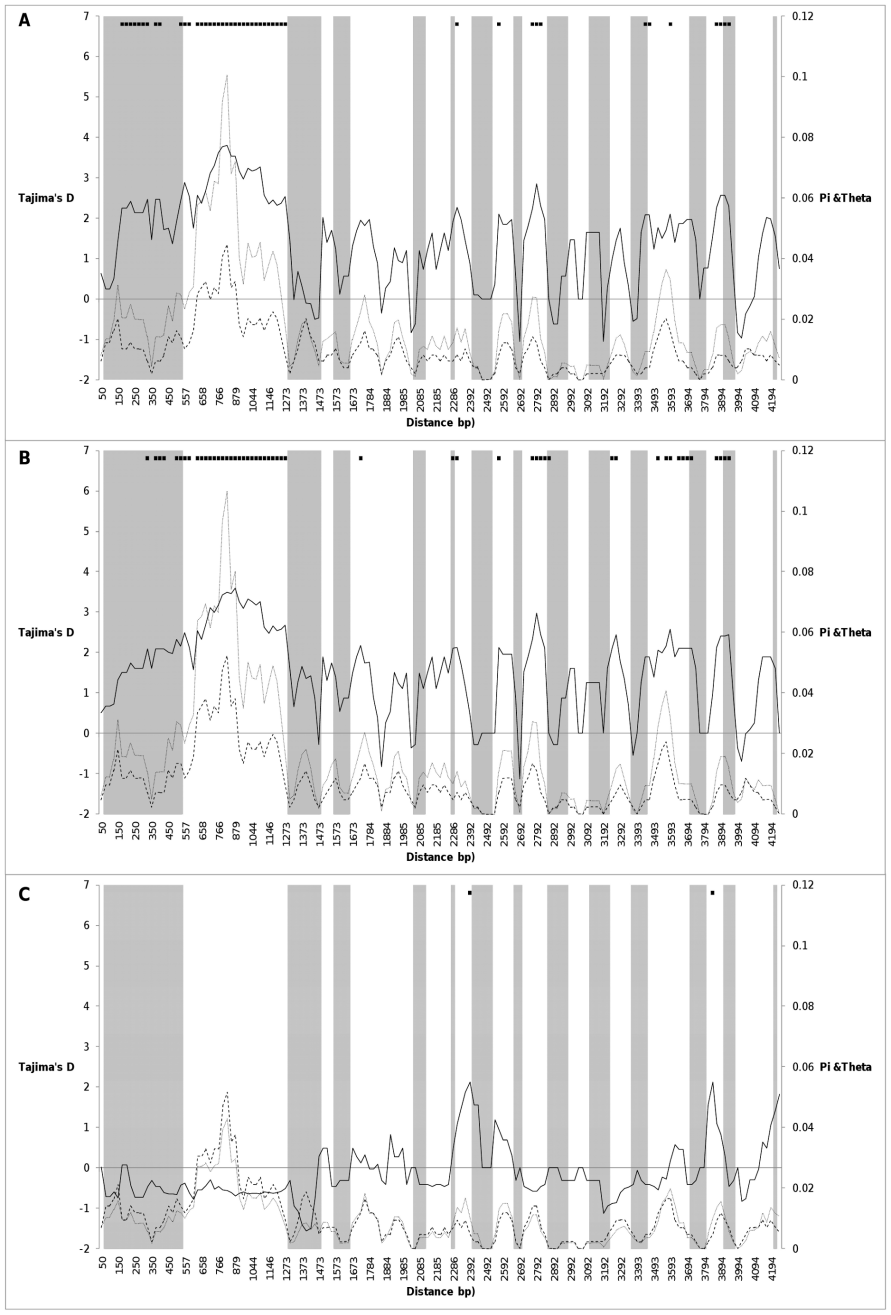
Overall nucleotide diversity (*π_T_*) (grey line), nucleotide polymorphism (*θ_w_*) (dashed line), and Tajima’s *D* value (black line), estimated along *CRTISO* gene for (A) the whole dataset, the (B) Western and (C) Eastern genetic groups. The analysis is based upon a 100 bp window with a step size of 25 bp. The exons and introns are shown as grey and white boxes, respectively. The significance for Tajima’s *D* is displayed at the top of each plot.

The overall nucleotide diversity was moderate (*π_T_* = 0.01753). The synonymous diversity value (*π_syn_* = 0.02788) was much higher than the non-synonymous one (*π_nonsyn_* = 0.00354), yielding a *π_nonsyn/_π_syn_* ratio of 0.12697. The nucleotide diversity was higher within non-coding regions (*π_non-coding_* = 0.02433) than within coding regions (*π_coding_* = 0.00928) (Table 4). High diversity values were observed within most of the introns, and particularly within intron 1 of *CRTISO* sequence (Figure 5A).

Tajima’s *D* statistic detected an excess of intermediate-frequency variants in the whole dataset, which yielded a significant positive *D* value (*D* = 2.76, *P*<0.01). High and significant *D* values were also obtained within non-coding regions (Table 5, Figure 5A). These *D* values were significant according to demographic models with bottleneck intensity *k* ≥10 (Figure S3).

**Table 5:** Tajima’s *D* value calculated for the whole dataset, the genetic and colour groups.

	No. of sequences	Tajima’s *D* value		
		Total	Coding regions	Non-coding regions
**Total**	78	2.75530**	1.7608	3.002**
**West**	38	2.89351**	2.3006*	2.9841**
**East**	40	−0.34631	−0.7079	−0.2502
**White**	12	1.95597	1.2712	2.0837*
**Yellow**	16	3.08636***	2.8237**	3.0579***
**Orange**	24	2.18514*	2.0361*	2.1500*
**Red**	12	−0.00587	−0.0431	−0.0343
**Purple**	14	−0.32423	−0.5050	−0.2982

* *P*<0.05;

** *P*<0.01;

*** *P*<0.001.

#### Within and between groups

Only the Western genetic group showed a similar pattern to the whole dataset for nucleotide diversity indexes and neutrality test (Tables 4 and 5; Figures 5A and 5B). The high nucleotide diversity observed within this genetic group was consistent with a balancing selection revealed by a positive and significant *D* value. This balancing selection hypothesis is reinforced by significance according to demographic models with *k* ≥7 (Figure S3).

Otherwise, the Eastern genetic group showed low nucleotide diversity (Table 4, Figure 5C) and a non significant negative Tajima’s *D* (Table 5).

Among the colour groups, the overall nucleotide diversity (*π_T_*) varied from 0.01105 to 0.02071, with an average value of 0.01614. The white-, orange- and yellow-rooted carrots showed globally higher nucleotide diversity than the red and purple ones (Table 4). These results were consistent with the detected selection pattern. Indeed, the yellow and orange carrots presented a positive *D* value for both coding and non-coding regions revealing a balancing selection, whereas the white type presented a significant one only for the non-coding region. These results were significant for demographic models with *k* ≥2 for yellow carrots, *k* ≥15 for orange carrots and *k* ≥7 for white carrots (Figure S3). Notice that the red and purple types showed a trend of negative Tajima’s *D* values even if they did not reach statistical significance (Table 5).

### Relationships among Haplotypes

**Figure 6:**
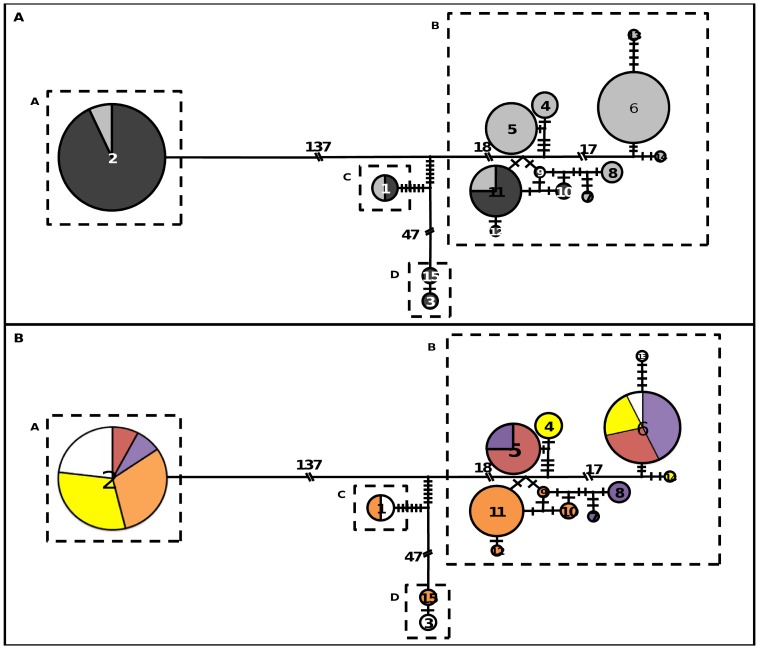
Median joining network derived from reconstructed DNA sequence haplotype of *CRTISO* gene. Haplotypes are displayed at network nodes and are symbolised by circles whose diameter is proportional to the frequency in the sample. The code number of the haplotypes is written in the circle centre. Perpendicular hashes on the lines joining haplotypes represent substitutions. When too numerous, they are displayed as slanting hashes with the number of substitutions differentiating two network nodes. The main clusters are identified by dotted boxes. (A) The black and grey proportions of each circle represent respectively the proportion of individuals from the Western and Eastern genetic groups (estimated by STRUCTURE analysis) sharing the same haplotype. (B) Colours represent the proportion of different colour groups: white, yellow, orange, red and purple.

The haplotype network revealed four main clusters (Figure 6A). The cluster A was the most distant one with 137 substitutions from the closest cluster. Based on haplotype 2 only, it contained mainly individuals belonging to the Western genetic group with white, yellow or orange roots (Table 1 and Figure 6B). The cluster B corresponded to 11 haplotypes. It contained yellow-, red- and purple-rooted carrots (and one white) belonging to the Eastern genetic group, and orange ones belonging predominantly to the Western genetic group but cultivated in Japan or Central Asia. In this cluster, all the orange Japanese material (haplotypes 9 to 12 only) was separated from other Asian cultivars.

Finally, the clusters C (only haplotype 1) and D (haplotypes 3 and 15) corresponded only to four individuals, with white or orange roots. Three out of four individuals belonged to the Western genetic group, whereas the two clusters were overall closer to the cluster B. The clusters C and D could constitute intermediate clusters.

## Discussion

### Variation of Polymorphism along the Complete Gene Sequence

In the present study, both results from microsatellites and *CRTISO* gene showed that cultivated carrot was divided into two genetic groups: one from Asia (Eastern genetic group), and another one mainly from Europe (Western genetic group). This subdivision is consistent with previous studies about microsatellite markers or genes [8], [9] and with the historical mention of two independent migration events from the domestication centre in Afghanistan: the first one to West in the 1200 s, and the second one to East in the 1400s [5], [6]. The haplotype network of *CRTISO* was especially marked by the genetic subdivision between the Western and Eastern genetic groups.

No decay of linkage disequilibrium was detected within 4,234 bp for *CRTISO* gene. This was consistent with results obtained by [8] which did not detect any decay of LD within 700–1,000 bp for some carotenoid biosynthetic genes in cultivated carrot. This result is unexpected for an allogamous species like carrot [49]. Indeed LD is known to decay quicker in outcrossing species than in selfing species [40]. For example, LD decays within 1,500 bp in maize [48], 200 bp in *Populus tremula* [50] or 500 bp in ryegrass [51]. Nevertheless, different factors could explain the observed high LD level such as low recombination rate, selection, and demographic events like population bottlenecks or population subdivisions.

By considering population differentiation obtained here, LD remained high in the Western and Eastern genetic groups, suggesting the population subdivision could not explain the absence of LD decay. However, *CRTISO* gene was mapped on the linkage group 4 near the centromeric region [27], and some studies revealed a high LD level in centromeric regions due to low recombination rate for several species (maize [48], human [52], [53]). Carrot is a recently domesticated and biennial species: this has resulted in fewer recombination events than expected. Despite the absence of LD decay in *CRTISO* gene, the Tajima’s *D* value varied a lot along the sequence, varying from significant to non significant values, especially in the whole dataset and the Western genetic group. The partial sequence analyzed in [8] and [25] showed a similar pattern than the complete sequence. However, caution is needed when conducting candidate-genes selection analysis on partial gene sequence.

### The Effect of Selection at *CRTISO* Gene

Deviations from neutrality expectations could be explained by selection or demographic events. The use of demographic models, considering both a moderate genetic bottleneck (*k* ≥2 to *k ≥*15, depending on the considered groups) during carrot domestication and a subsequent population subdivision between the Eastern and Western groups, confirmed the significance of observed *D* values. However, it should be noted that for an intense bottleneck hypothesis (*k* <2), the significance disappeared. Bottleneck intensity usually found for domestication ranges from *k = *0.2 (*Oryza sativa* ssp. *japonica* [54]) to *k* = 2.45 (maize [55]). These domestication events led a significant loss of diversity in these cultivated species by comparison to their wild relatives. By comparison, carrot may have experienced a weaker domestication bottleneck because no significant difference of genetic diversity level was found in cultivated carrot by comparison to wild carrot [56]. Therefore, it is unlikely that the observed significant neutrality tests were obtained only by population size variations during domestication or by population subdivision during cultivation history. These simulations give credit to the selection hypothesis.

The possibility of photoisomerisation in leaves made us hypothesize that *CRTISO* gene may have evolved with less constraints and may have been more prone to artificial selection, for example for root colour. Indeed, the analysis of the complete *CRTISO* sequence demonstrated a significant positive Tajima’s *D* value, which indicates a departure from the neutral expectation. This significant positive value was observed for the whole dataset and the Western genetic group, whereas the Eastern genetic group exhibited a neutral evolution pattern. Therefore it appears that *CRTISO* evolved differentially in the Western and Eastern genetic groups.

Indeed, these results suggest balancing selection as the force governing *CRTISO* evolution in the Western genetic group, and are congruent with the high linkage disequilibrium and the high silent-site nucleotide diversity detected in this genetic group. Furthermore, the results obtained in our study are in agreement with the previous results [8], [25] based on the analysis of a partial *CRTISO* sequence corresponding mainly to the intron 1. By contrast, the Eastern genetic group showed no selection with a low nucleotide diversity.

One explanation for the potential balancing selection observed in the Western genetic group would be the selection for some colour types. A signature of balancing selection was found at *CRTISO* gene within yellow and orange carrots (*D* = 3.08636, *P*<0.001; *D* = 2.18514, *P*<0.05, respectively). Several models of selection maintaining diversity were suggested: heterozygote advantage at a locus, frequency-dependent selection, temporally or spatially heterogeneous selection [57]. The latter hypothesis is the most plausible for carrot colour selection. Carrot has been bred for new colours between the domestication of this species and the eighteenth century, and these selective events occurred in the centre of origin (Afghanistan region), in Europe and in Asia. Therefore, the breeding objectives varied temporally and spatially for carrot root colour.

However, caution is needed while interpreting the *CRTISO* polymorphism in yellow and orange carrots as evidence for signature of selection. Indeed, the differentiation between colour groups was considerably influenced by the subdivision between the Western and Eastern genetic groups. White carrots were mostly in the Western genetic group, four among six. Otherwise, all red and purple carrots (except the cultivar ‘Honbeni Kintoki’, code 426 unexpectedly) were assigned to the Eastern genetic group. The yellow group was half constituted by Western and Eastern individuals concordantly for microsatellite and *CRTISO* data, while the orange group was composed of individuals mainly assigned to the Western genetic group, with some individuals cultivated in Asia showing *CRTISO* haplotypes common to Eastern carrots. Therefore, these two colour groups are probably the most made of the two genetic groups. The respective effect of population subdivision, which could mimic balancing selection, and real balancing selection in these two colour types should be investigated in the future.

Selection footprint and high LD extent detected among *CRTISO* gene might lead to a selective sweep around this gene [58]. The low intrachromosomic LD observed in our study could not confirm this hypothesis. New markers in *CRTISO* genomic region could be developed thanks to carrot genome sequencing, and therefore could allow improving the study of selection events in this species.

### Insights into the History of Cultivated Carrots

Neutral loci and *CRTISO* polymorphism analyses allowed testing the hypotheses about cultivated carrot history. Microsatellite markers gave information about the entire genome and were therefore very suitable to understand gene pool origin. The incongruity between the *CRTISO* haplotype network and genetic structure given by microsatellite analysis informed about genetic admixture.

Literature considers the purple and yellow carrots as the two original colour types [4], [5]. The purple type mainly found in Middle East was clearly assigned to the Eastern group, both from microsatellite and *CRTISO* sequence data. By comparison, the clustering of yellow cultivars half into the Western group and half into the Eastern group, whether through microsatellite or *CRTISO* sequence analyses, suggested that the yellow type had experienced two separate migration events, following domestication.

According to their assignment probability and large confidence interval estimated by STRUCTURE analysis, orange carrots sampled from Japan and Central Asia were assigned to the Western genetic group but with some admixture between Western and Eastern carrots whereas they belonged clearly to the Asian cluster depending on *CRTISO* data. This is congruent with the breeding of this colour type. Indeed some European cultivars were exported and adapted for cultivation to Japan in the 1900 s. They were used in breeding programs and crossed with Asian carrots [59]. In the same way, carrots from Central Asia might come from European cultivars that were either exported and cultivated straight in Central Asia, or went by Japan before being used in Central Asia. Two out of three were significantly assigned to the Western genetic group based on the microsatellite results, but to the Asian cluster and an intermediate one (haplotype 15) based on *CRTISO* data. It remains therefore unclear if Central Asia cultivars came from European material crossed locally with Eastern material, or they originated from an Eastern migration from Japan.

## Conclusions

Neutral loci and *CRTISO* polymorphism analyses were shown to be complementary in order to recount the breeding history of cultivated carrot. Indeed, human activities (seed exchange and transport, and breeding) could affect allele diversity, which resulted in the distinction of two genetic groups and large diversity at the *CRTISO* gene. These results may suggest that human preferences for carrot root colour have varied depending on periods and geographical areas. It brings new insights about the history of orange carrots. The orange-rooted carrots spread from Europe to other continents including Asia. Therefore the breeding of this carrot form was adapted to different markets. In spite of nucleotide polymorphism and the distinction of several haplotype clusters within the orange carrots, corresponding individuals belonged mainly to the Western genetic group based on microsatellite data. This would confirm that the introduction of European carrots in Asian breeding programs is relatively recent, as genetic background remains common to both plant materials. The knowledge of the breeding history of this species, based both on neutral and functional loci, allows a better characterisation of plant material, and offers substantial insight to secure, manage and exploit carrot genetic resources while maximising genetic diversity.

## Supporting Information

Figure S1 **Plots of (A) Delta K and (B) the log likelihood, from the STRUCTURE analysis.**
(TIF)

Figure S2 **Genetic structure of 39 carrot cultivars based on a correspondence analysis (CA) on microsatellite data.** Squares and circles represent respectively individuals from the Western genetic group and individuals from the Eastern genetic group according to STRUCTURE results.
(TIF)

Figure S3 **Significance of Tajima’s** ***D*** **according to the genetic bottleneck severity** ***k*** **, for (A) the total sequence, (B) the coding sequence and (C) the non-coding sequence.** Ten values of bottleneck severity from 0.2 (most severe) to 50 (least severe) were tested. The significance was displayed for the whole dataset (black line), Western group (blue line), Eastern group (green line), white group (grey line), yellow group (yellow line), orange group (orange line), red group (red line) and purple group (purple line). The significance is shown as *P*-values for two-tailed Tajima’s test, with a logarithmic scale (*y*-axis). *P*-values were calculated by comparing the location of observed Tajima’s *D* with the simulated dataset obtained by the demographic model. In each plot, the areas with *P*<0.001, *P*<0.01 and *P*<0.05 are represented with red, orange and yellow backgrounds, respectively.
(TIF)

Table S1 **Characteristics of 17 carrot microsatellite markers** [**27**] **.**
(XLSX)
