# Supplementary figures and images for: Functional Gene Polymorphism to Reveal Species History: The Case of the CRTISO Gene in Cultivated Carrots
Source: PLoS One. 2013 Aug 5;8(8):e70801. doi: 10.1371/journal.pone.0070801 (PMC3733727; doi:10.1371/journal.pone.0070801)

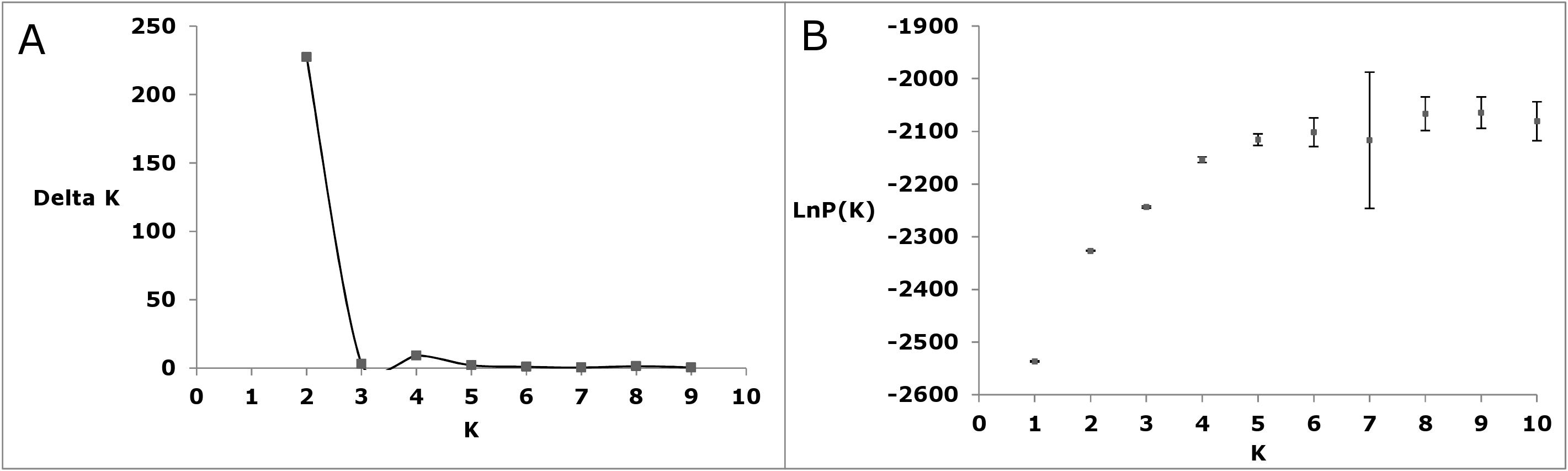

Supplement: Figure S1 — Plots of (A) Delta K and (B) the log likelihood, from the STRUCTURE analysis. (TIF) [file pone.0070801.s001.tif]

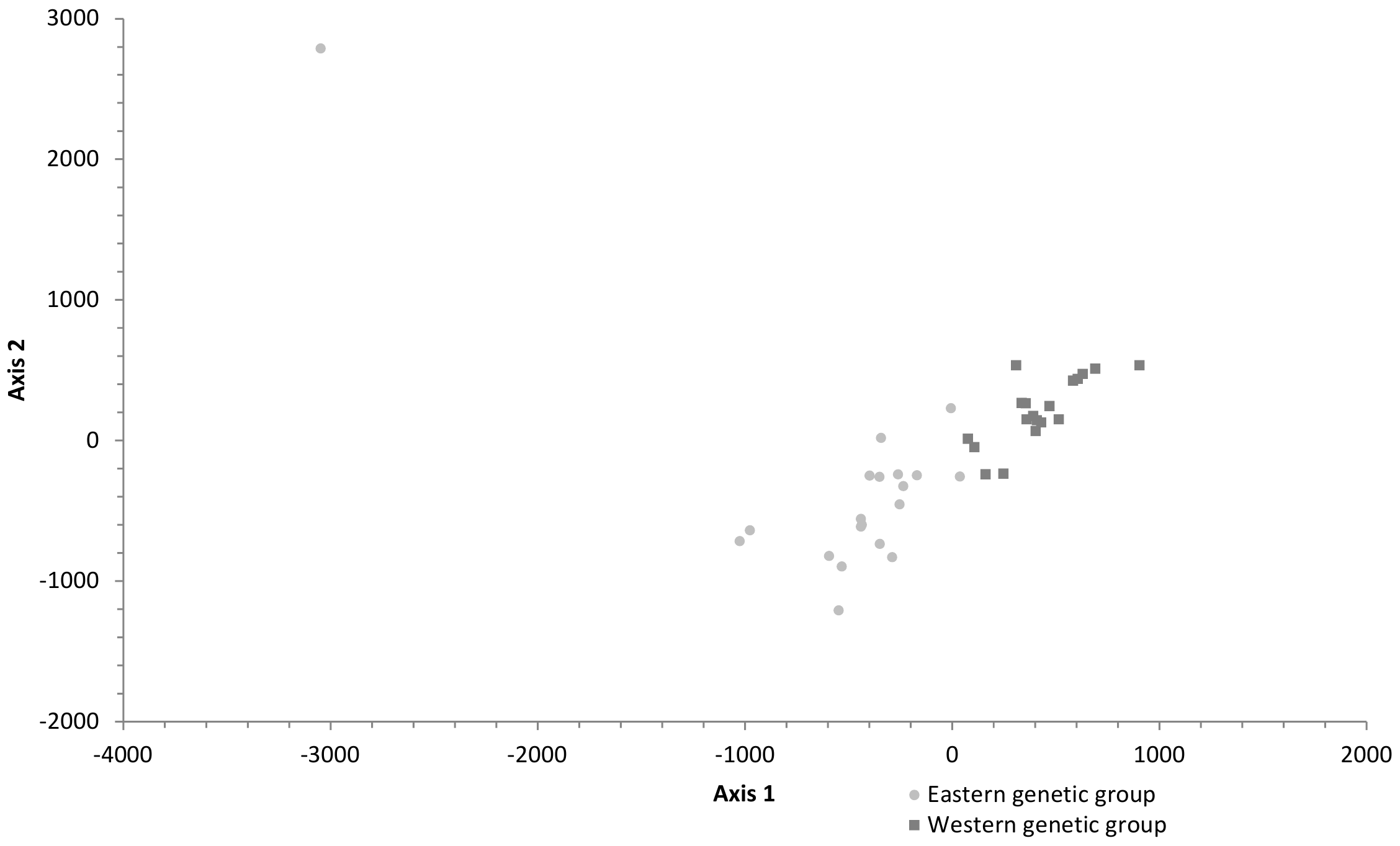

Supplement: Figure S2 — Genetic structure of 39 carrot cultivars based on a correspondence analysis (CA) on microsatellite data. Squares and circles represent respectively individuals from the Western genetic group and individuals from the Eastern genetic group according to STRUCTURE results. (TIF) [file pone.0070801.s002.tif]

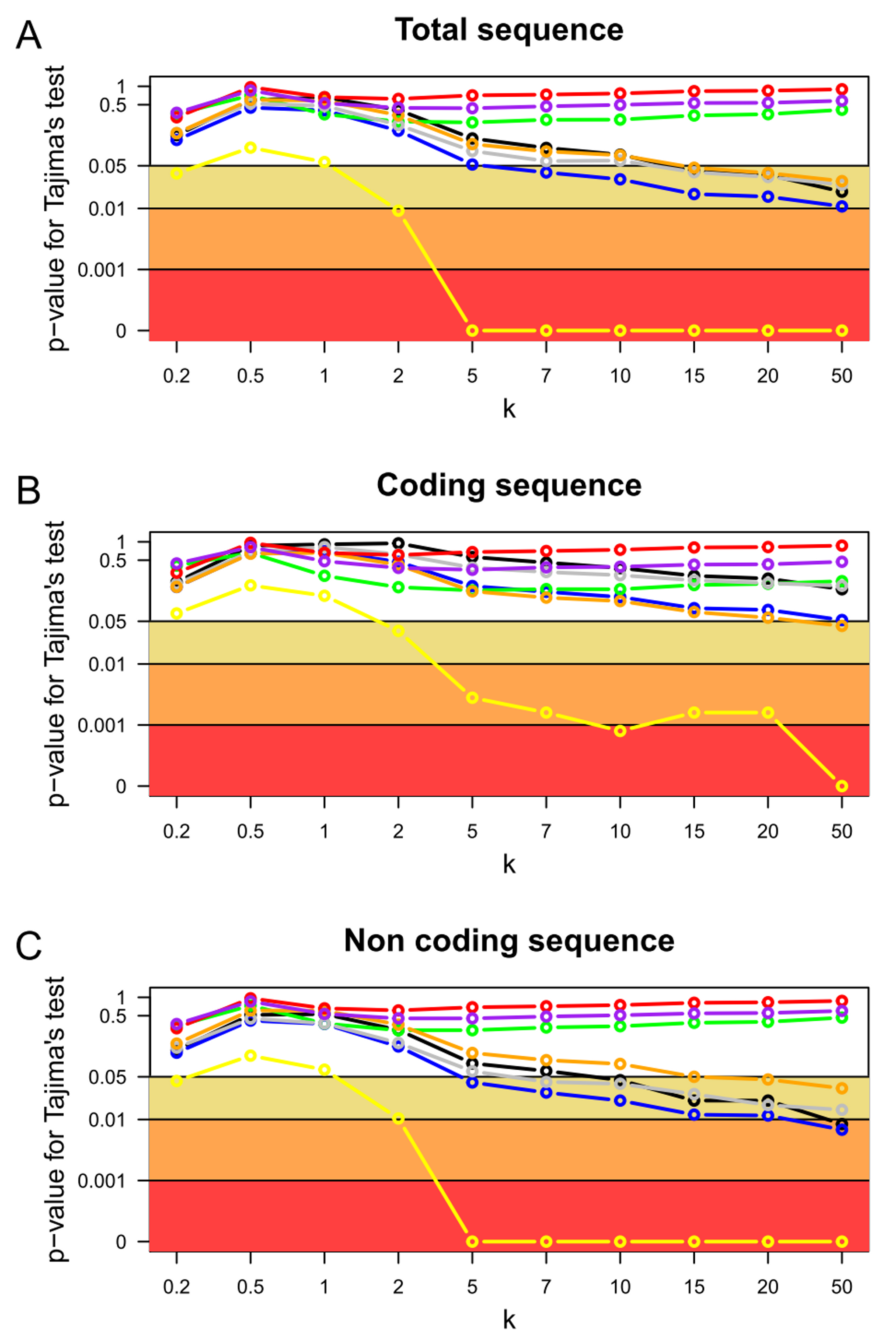

Supplement: Figure S3 — Significance of Tajima’s D according to the genetic bottleneck severity k , for (A) the total sequence, (B) the coding sequence and (C) the non-coding sequence. Ten values of bottleneck severity from 0.2 (most severe) to 50 (least severe) were tested. The significance was displayed for the whole dataset (black line), Western group (blue line), Eastern group (green line), white group (grey line), yellow group (yellow line), orange group (orange line), red group (red line) and purple group (purple line). The significance is shown as P-values for two-tailed Tajima’s test, with a logarithmic scale (y-axis). P-values were calculated by comparing the location of observed Tajima’s D with the simulated dataset obtained by the demographic model. In each plot, the areas with P<0.001, P<0.01 and P<0.05 are represented with red, orange and yellow backgrounds, respectively. (TIF) [file pone.0070801.s003.tif]
